# Leaky pipeline, gender bias, self-selection or all three? A quantitative analysis of gender balance at an international palliative care research conference

**DOI:** 10.1136/bmjspcare-2016-001211

**Published:** 2017-03-07

**Authors:** Katherine E Sleeman, Jonathan Koffman, Irene J Higginson

**Affiliations:** Department of Palliative Care, Policy and Rehabilitation, King's College London—Cicely Saunders Institute, London, UK

**Keywords:** gender balance, leaky pipeline, palliative

## Abstract

**Objectives:**

The ‘leaky pipeline’ in academia is a clearly described phenomenon, but has not been examined in palliative care. We analysed the gender balance of speakers at the 9th World Research Congress of the European Association of Palliative Care (EAPC) to test the null hypothesis that there is no difference in the proportion of women and men with senior academic visibility in palliative care conference programmes.

**Methods:**

The final programme of the 2016 EAPC World Congress was examined, and the gender of each speaker was recorded. Presentations were assessed using a three-tier hierarchy of senior academic visibility: Free Communication sessions, Themed sessions and invited Plenaries (low to high). As there was only one Invited Plenary at EAPC 2016, we examined the gender balance at EAPC Plenaries from 2012 to 2016.

**Results:**

Overall, the majority of speakers at EAPC 2016 (96/130, 73.8%) were women. The proportion of women was highest in the Free Communication sessions (84/107, 78.5%). In the Themed sessions, women made up just over half of speakers (12/22, 54.5%). In 2016, there was 1 invited Plenary speaker, a man. From 2012 to 2016, just 6 of 23 invited Plenary speakers at EAPC conferences have been women (26.1%) (χ^2^=25.4, p<0.001).

**Conclusions:**

These data reject our null hypothesis and suggest that there is attrition of women along the academic pipeline in palliative care. Other factors such as self-selection (that women decline invitations to give talks) and unconscious gender bias need further exploration, as well as actions to address the imbalance.

## Introduction

The gender balance in academia is the subject of considerable debate.^1–3^ The progress of women through academia has been described as a ‘leaky pipeline’, where there is attrition of women at each step of the career ladder. Initiatives to redress this are gaining momentum, for example the UK Athena SWAN Charter which was established in 2005 to encourage and recognise commitment to advancing the careers of women in science, technology, engineering, maths and medicine (STEMM).

Visibility, for example through conference talks and plenaries, is an important dimension of gender equality. A paucity of women conference speakers has been demonstrated in several academic disciplines.^4^ However, it is unclear if the same phenomenon exists in palliative care, a specialty where women traditionally make up the majority of the workforce.^5^

In June 2016, 1200 delegates attended the 9th World Research Congress of the European Association of Palliative Care (EAPC) in Dublin, making it one of the largest research conferences in palliative care globally. The programme featured 17 Free Communication sessions where researchers were chosen on the quality of anonymous (and gender-blind) abstracts to present their work in 8 min talks. In addition, there were seven Themed sessions in which speakers were invited on the basis of their level of authority on a specific subject to give 20 min talks. The most prestigious speaking opportunity is the invited Plenary where high-profile academics are invited to give longer talks (30 min) on the main conference stage.

We aimed to analyse the gender balance of speakers at the 2016 EAPC World Congress to test the null hypothesis that there is no attrition of women in senior academic positions in palliative care, using the three-tiered hierarchy of Free Communication sessions, Themed sessions and Plenaries as a proxy for seniority.

## Methods

The final programme of the 2016 EAPC World Congress was examined, and the gender of each speaker was recorded. Speaker gender was determined either by direct observation of their talk or by inference from their first name. In eight cases, Google was used for more information. Where there was a substitution of speaker at the last minute, the gender of the speaker originally planned was used. At the 2016 EAPC, five top scoring abstracts were presented as 15 min Plenaries on the main stage. Since the decision to feature these presentations was made on the basis of an anonymous abstract, these were analysed with the Free Communication sessions. Since there was only one invited Plenary speaker at 2016 EAPC, we examined the gender balance of invited EAPC Plenary speakers over 5 years (2012–2016) by searching through relevant scientific programmes online. χ^2^ tests were used to assess differences. We did not analyse Meet the Expert sessions or Poster Discussion sessions.

## Results

At EAPC 2016, there were a total of 17 Free Communication sessions comprising 102 talks. An additional five top scoring abstracts were presented on the main stage. There were seven Themed sessions which included 22 speakers. There was a single invited Plenary, the Ventafridda Lecture.

Overall, the majority of speakers at EAPC 2016 were women. The proportion of women was highest in the Free Communication sessions. In the Themed sessions, women made up just over half of speakers. The Ventafridda Lecture was given by a man (table 1).

**Table 1:** Men and women giving talks at EAPC 2016, according to session type

	Men (%)	Women (%)
Invited plenary	1 (100)	0 (0)
Themed session	10 (45.5)	12 (54.5)
Free communication	23 (21.5)	84* (78.5)
Total	34 (26.2)	96 (73.8)

*Includes five top scoring abstracts presented on the main stage.

**Figure 1:**
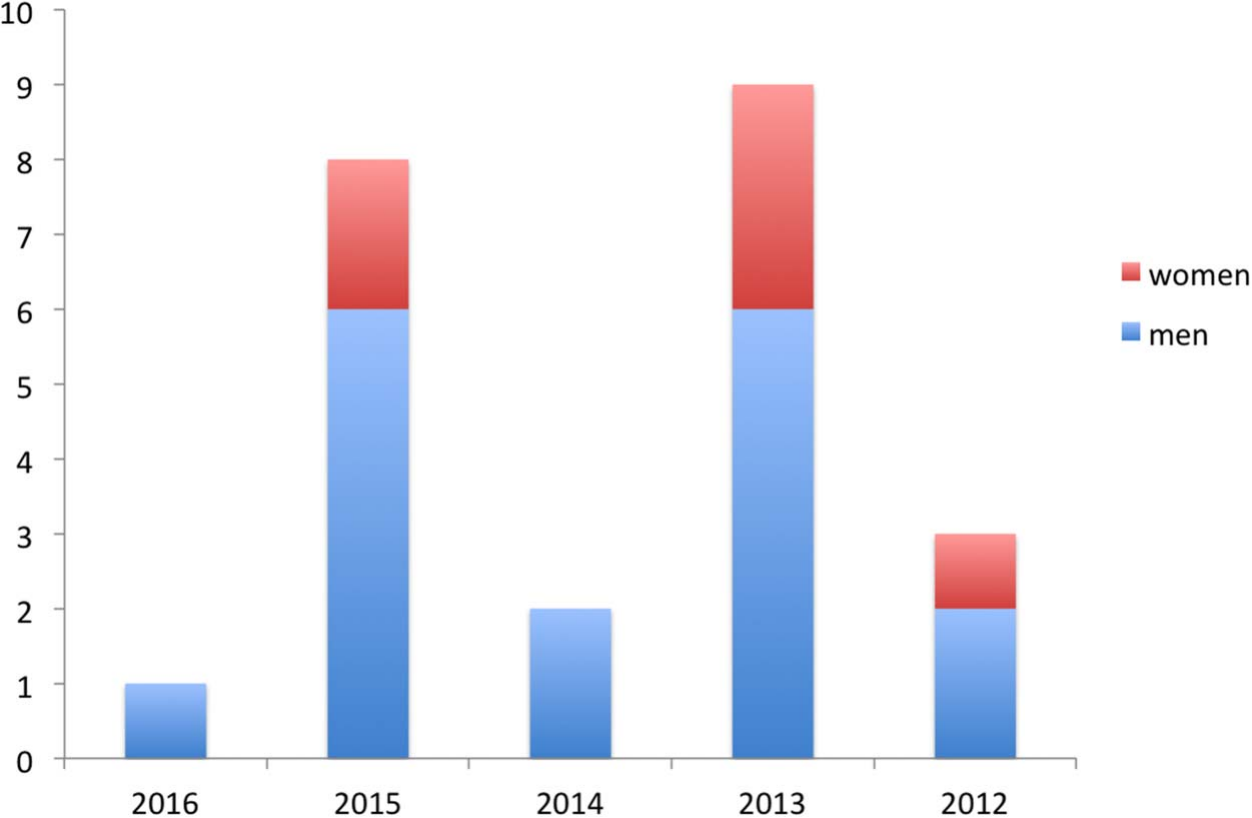
Number of men and women giving invited Plenaries, 2012–2016.

Since there was only one invited Plenary speaker at EAPC 2016, we examined previous EAPC conference programmes to obtain a more representative view of gender balance of invited Plenary speakers for a 5-year period (figure 1). At EAPC 2015, there were eight invited Plenary speakers (including a debate), of whom two were women (25%). At EAPC 2014, there were two Plenary speakers, both men. At EAPC 2013, there were nine Plenary speakers, including three women (33.3%). At EAPC 2012, one of three Plenary speakers was a woman (33.3%). Taking 5 years together, 6 of 23 invited Plenary speakers at EAPC conferences have been women (26.1%) (χ^2^=25.4, p<0.001).

## Discussion

Palliative care is a profession where women make up the majority of the workforce,^5^ and this is reflected in the overall gender balance of those giving talks at EAPC 2016. However, we identified that the proportion of women is lower for invited talks compared to those chosen anonymously from abstracts: whereas around three quarters of speakers in the Free Communication sessions were women, this proportion reduced to roughly half for the Themed sessions, and to only a quarter for the invited Plenaries (using pooled data from 2012 to 2016).

Given the large number of women working in palliative care, it is of concern that the most prestigious invited Plenaries are so infrequently given by women, and it is noteworthy that two EAPC conferences over the past 5 years have included no female Plenary speakers at all (2014 and 2016). These data suggest that there is attrition of women along the ‘academic pipeline’ in palliative care. However, the relative contribution of other potential factors such as self-selection (that women decline invitations to give talks) and unconscious gender bias (that women are not invited to give talks) is unclear. In addition, palliative care is multiprofessional, with gender unequally represented within each of the professions. The potential contribution of discipline should also be explored.

Given that there was a single Plenary speaker in 2016, we examined the gender balance of Plenary speakers over 5 years. This was based on the assumption that variation from conference to conference is likely to outweigh any temporal trends. The proportion of female Plenary speakers was one-third or fewer for each of the 5 years examined. There was no indication that the proportion of female Plenary speakers improved over time. Indeed, the 2012 and 2013 conferences had the greatest proportion of female Plenary speakers (one-third).

Speaking at academic conferences is important for career advancement. Such opportunities not only facilitate networking and collaboration, but are used as a marker of quality by promotions panels.^6^ Strategies to improve the proportion of women invited to speak, particularly those invited to give Plenaries, should be explored. These include consideration of the gender balance on scientific organising committees, as the proportion of women on convening panels has been shown to be correlated with the proportion of women speakers.^4^ ^6^ The issue of self-selection is important: previous studies have shown that women are more likely than men to decline invitations to speak at conferences,^7^ and less likely than men to put themselves forward for talks.^8^ These issues should be explored and addressed, for example through routine collection and analysis of the gender of all authors who submit abstracts, not only for EAPC Congresses but for all major palliative care conferences. Scientific organising committees should scrutinise information on invitations, acceptances and refusals for high-profile speaking positions. Where women decline invitations to speak, the reasons for this should be explored, and steps to increase future acceptance rates identified. These initiatives will help us to understand the relative contributions of lack of assertiveness and lack of recognition. In addition, routinely collecting feedback from delegates on the issue of gender equality might identify hidden issues. Last, we suggest that explicit acknowledgement of low visibility of female speakers by conference organisers may itself act as an intervention to redress the balance.

Failure of promotion on merit is deleterious for academia and ultimately for patient care. Whether these data represent a leaky pipeline, gender bias, self-selection or all three, it is essential that as a specialty we take this issue seriously. We hope that the data presented here provide a starting point for more in-depth consideration of this issue and appropriate strategies to tackle it.
